# Sex differences in clinical and imaging characteristics of axial juvenile spondyloarthritis

**DOI:** 10.1093/rheumatology/keaf611

**Published:** 2026-03-03

**Authors:** Adam S Mayer, Rui Xiao, Timothy G Brandon, Pamela F Weiss, Juvenile Axial Spondyloarthritis Research Taskforce (JAxSpRT), Amita Aggarwal, Ruben Burgos-Vargas, Robert A Colbert, Gerd Horneff, Ronald M Laxer, Kirsten Minden, Angelo Ravelli, Nicolino Ruperto, Judith A Smith, Matthew L Stoll, Shirley M Tse, Filip Van den Bosch, Walter P Maksymowych, Robert G Lambert, David M Biko, Nancy A Chauvin, Michael L Francavilla, Jacob L Jaremko, Nele Herregods, Ozgur Kasapcopur, Mehmet Yildiz, Hemalatha Srinivasalu, Daniel J Lovell, Peter A Nigrovic, Ivan Foeldvari, Marisa S Klein-Gitelman, Seza Ozen, Ray Naden, Alison M Hendry, Rik Joos

**Affiliations:** Divisions of Adult and Pediatric Rheumatology, Hackensack University Medical Center, Hackensack, NJ, USA; Hackensack Meridian School of Medicine, Nutley, NJ, USA; Department of Biostatistics, Epidemiology and Informatics, Perelman School of Medicine, University of Pennsylvania, Philadelphia, PA, USA; Department of Pediatrics, Division of Biostatistics, Children’s Hospital of Philadelphia, PA, USA; Department of Pediatrics, Division of Rheumatology, Children's Hospital of Philadelphia, PA, USA; Clinical Futures, Children’s Hospital of Philadelphia, Philadelphia, PA, USA; Department of Pediatrics, Division of Rheumatology, Children's Hospital of Philadelphia, PA, USA; Clinical Futures, Children’s Hospital of Philadelphia, Philadelphia, PA, USA; Center for Clinical Epidemiology and Biostatistics, Perelman School of Medicine, University of Pennsylvania, Philadelphia, PA, USA

**Keywords:** axial spondyloarthritis, sex, paediatrics

## Abstract

**Objectives:**

The impact of biologic sex in axial juvenile spondyloarthritis (axJSpA) is unknown. We assessed whether biologic sex is associated with disease manifestations, patient-reported outcomes, or characteristic sacroiliac joint (SIJ) MRI lesions in a large cohort of youths with classified axJSpA.

**Methods:**

This international multicentre cross-sectional study included youths aged <18 years with physician-diagnosed juvenile spondyloarthritis and fulfilling the classification criteria for axJSpA. Clinical and SIJ MRI data were available from the time axial disease was first diagnosed and were compared between males and females using Pearson’s chi-squared and Wilcoxon rank-sum tests, as appropriate. Multivariable logistic regression evaluated the association of sex with inflammatory and structural MRI lesions typical of axial disease.

**Results:**

Among the 143 patients included, 67.1% were male. Males had significantly greater HLA-B27 positivity, hip/groin stiffness and inflammatory marker elevation. There were no differences in peripheral arthritis, enthesitis or patient-reported outcomes. On SIJ MRI, males had significantly higher odds of unequivocal inflammatory lesions (OR 4.86, 95% CI 1.37–17.32), bone marrow oedema (OR 4.13, 95% CI 1.17–14.62) and pelvic enthesitis (OR 5.23, 95% CI 1.39–19.61) compared with females, but no differences in structural lesions were found.

**Conclusion:**

In a large multicentre axJSpA cohort, males were significantly more likely to have clinical and MRI features of inflammatory sacroiliitis at the time axial disease was first diagnosed. Future trials in axJSpA should strongly consider stratification by sex in their design.

## Graphical Abstract

**Figure:**
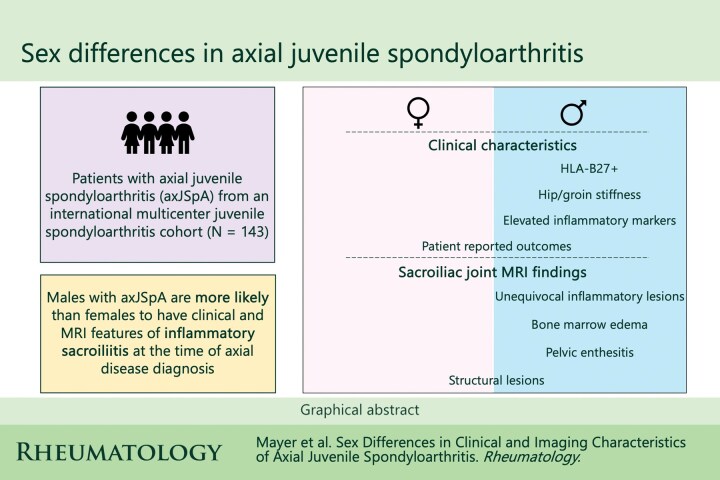

Rheumatology key messages

- Males with axJSpA had significantly higher odds of unequivocal inflammatory MRI lesions and pelvic enthesitis.
- There were no sex differences in peripheral arthritis, enthesitis or patient-reported outcomes.
- Future trials in axJSpA should strongly consider stratification by sex in their design.

## Introduction

Biologic sex is increasingly appreciated as having important implications in the manifestations and treatment of adult axial spondyloarthritis (axSpA). The pathophysiology underlying these sex differences remains poorly understood, with hypothesized roles for sex-dependent immunologic, genetic and hormonal pathways [1–3]. For example, the administration of oestrogen to ovariectomized SKG mice has been found to suppress inflammatory arthritis and decrease expression of cytokines important in spondylarthritis pathogenesis, such as those on the interleukin-17 axis [1]. Over the past decade, key differences in clinical characteristics, radiologic findings and patient-reported outcomes have been identified between adult male and female patients with axSpA [4–12]. Male sex is a known risk factor for both inflammatory sacroiliitis and radiographic progression in the sacroiliac joint (SIJ) [4, 5, 13, 14] while females tend to have worse patient-reported outcomes and functional status [4, 8–10, 12, 15–17]. However, the existence of such sex differences in paediatric cohorts has never been explored and may plausibly differ from those in adults, especially given differences in the classification of axial disease in the two populations [18, 19].

While an evaluation of sex differences in clinical characteristics and patient-reported outcomes is needed, differences in inflammatory and structural lesions in the SIJ on MRI are particularly important as these imaging findings are considered necessary for both the clinical diagnosis and classification of axial disease in juvenile spondyloarthritis (JSpA) [18, 20]. Additionally, inflammatory SIJ lesions are anatomically associated with the occurrence of local structural damage over time [21] and thus are a critical factor to assess in a young population who will live for decades with this chronic immune-mediated disease. Ultimately, an assessment of the association between biologic sex and clinical and imaging characteristics in patients with axial juvenile spondyloarthritis (axJSpA) is both clinically important and will lead to a better understanding of how to handle biologic sex as a covariate in trials of axJSpA.

The objective of this study was to determine whether biologic sex is associated with disease manifestations, patient-reported outcomes or the presence of inflammatory and/or structural MRI lesions in the SIJ at the time axial disease was initially diagnosed in a large international multicentre cohort of youths with classified axJSpA.

## Methods

This study was reviewed by the Children’s Hospital of Philadelphia (CHOP) Institutional Review Board (IRB), and the IRB determined the procedures met the exemption criteria per 45 Code of Federal Regulations 46.104(d) 4(iii) (IRB19-016078).

### Study design and patient selection

This study is a secondary analysis of data from an international multicentre cross-sectional study of a cohort of patients who met axJSpA criteria that have previously been published [18]. Details on methodology and data collection have been previously described [18]. The axJSpA classification score ranges from 0 to 100 with a classification threshold of ≥55 and is comprised of six domains: unequivocal evidence of inflammatory or structural lesions on MRI of the SIJs, pain chronicity, pain pattern, pain location, morning stiffness and genetics (HLA-B27 positivity in patient or first-degree relative). This study included the youths from that cohort age <18 years with a physician diagnosis of JSpA who fulfilled classification criteria for axJSpA.

Cross-sectional data on clinical characteristics, including demographics, symptoms, exam findings, laboratory values, MRI and patient/physician-reported outcomes were obtained from the clinical visit at which axial disease was diagnosed. Current use of medications known to treat axial disease was also available, including NSAIDs, tumour necrosis factor alpha inhibitors (TNFi), interleukin-17 inhibitors (IL-17i) and Janus kinase inhibitors (JAKi). At least three members of a central imaging team with expertise in musculoskeletal imaging completed the MRI assessment for each patient. Imaging experts reviewed MRI studies independently and were blinded to clinical details other than age and sex.

### Exposure and outcomes

The primary exposure of interest was biologic sex. The co-primary outcomes were the presence on MRI of (i) unequivocal SIJ inflammatory lesion(s), defined as bone marrow oedema in ≥3 quadrants across all SIJ slices [22] and (ii) unequivocal SIJ structural lesion(s), defined as erosion in ≥3 quadrants or sclerosis or fat lesion in ≥2 SIJ quadrants or backfill or ankylosis in ≥2 joint halves across all SIJ slices and structural MRI lesions [22]. Secondary outcomes included component inflammatory and structural MRI lesions as defined by the ASAS classification criteria for active sacroiliitis on MRI [23, 24] and the preliminary Juvenile Idiopathic Arthritis Magnetic Resonance Imaging Score—Sacroiliac Joint (JAMRIS-SIJ) from the Outcome Measures in Rheumatology (OMERACT) working group [25].

### Covariates

Several covariates of interest were included given their clinical relevance and known association with biologic sex in adult cohorts of recent-onset axial spondyloarthritis [4], including age, HLA-B27 status, first-degree relative with spondyloarthritis, past or current peripheral arthritis and inflammatory marker elevation (CRP or ESR normal *vs* abnormal).

### Statistical analysis

Clinical and imaging characteristics and patient/physician-reported outcomes were compared between male and female patients using Pearson’s chi-squared or Fisher’s exact test as appropriate for categorical variables and Wilcoxon rank-sum test for continuous variables. Separate multivariable logistic regression models were used to assess the association between inflammatory and structural MRI findings typical of axJSpA and sex. Candidate covariates with *P* < 0.2 in univariate logistic regression models were sequentially selected for the multivariable model using a stepwise model selection approach. Given the association of HLA-B27 positivity with both inflammatory and structural sacroiliac joint lesions in adult axSpA [26], the interaction between sex and HLA-B27 status was also tested in the primary analysis. Covariate selection and goodness-of-fit testing (Akaike and Bayesian information criteria) were conducted separately for the inflammatory and structural lesion models. Missingness was <16% across all covariates and addressed using multiple imputation by chained equations with 10 imputations. All statistical analyses were performed using Stata 17.0 (StataCorp LLC, College Station, TX, USA).

## Results

### Patient cohort

A total of 143 patients met inclusion criteria of which 96 (67.1%) were male, 64.0% were HLA-B27 positive and 18.1% had a family history of spondyloarthritis in a first-degree relative.

### Clinical characteristics

**Figure 1.**
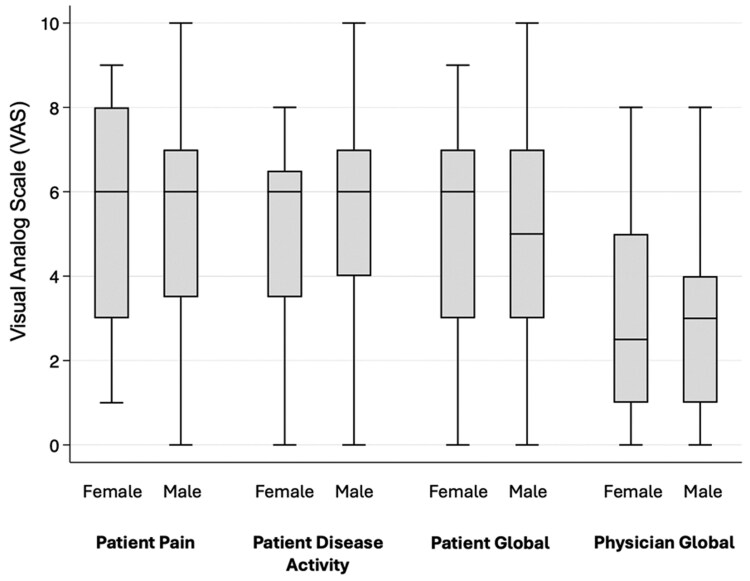
Differences in physician- and patient-reported outcomes between male and female patients with axial juvenile spondyloarthritis

Table 1 displays clinical characteristics of male and female patients. In comparison to females, male patients had significantly greater prevalence of HLA-B27 positivity (*P* < 0.01), hip/groin stiffness (*P* = 0.01) and elevated inflammatory markers (*P* = 0.03). The discrepancy in inflammatory marker elevation was driven by CRP (elevated in 63.9% of males *vs* 36.8% females) rather than ESR (elevated in 58.8% of males *vs* 52.5% females). There were no significant differences between males and females in prevalence of comorbid psoriasis or inflammatory bowel disease, enthesitis, peripheral arthritis, total body pain or use of NSAIDs or TNFi at the time axial disease was initially suspected. A majority of both male and female patients had symptom duration ≥12 weeks prior to axial disease evaluation and symptom duration did not significantly differ between groups (*P* = 0.24). Patient- and physician-reported measures also did not significantly differ between male and female patients (Fig. 1).

**Table 1.** Subjects

Feature	Female (*n* = 47) N (%) or median (IQR)	Male (*n* = 96) N (%) or median (IQR)	*P*-value
**Demographics and comorbidities**			
Age at axial disease evaluation (*n* = 142)	13.7 (12.0, 16.6)	15.2 (13.0, 17.0)	0.14
Body mass index	20.8 (17.6, 23.2)	20.2 (17.4, 23.6)	0.52
HLA-B27 (+) (*n* = 136)	20 (44.4)	67 (73.6)	<0.01
SpA in first-degree relative (*n* = 133)	12 (26.1)	12 (13.8)	0.08
History of psoriasis (*n* = 139)	1 (2.1)	2 (2.1)	1.00
History of IBD (*n* = 139)	5 (10.6)	9 (9.4)	0.92
**Patient-reported symptoms**			
Hip or groin pain (*n* = 141)	25 (54.3)	66 (69.5)	0.08
Hip or groin stiffness (*n* = 126)	12 (31.6)	49 (55.7)	0.01
Lumbar/sacral/buttock pain	37 (78.7)	75 (78.1)	0.93
Total body pain (*n* = 142)	0 0	1 (1.0)	0.49
**Arthritis/enthesitis history and/or exam**			
Enthesitis (*n* = 136)	16 (36.4)	45 (48.9)	0.17
Tender enthesis count	0 (0, 1)	0 (0, 1)	0.19
Peripheral arthritis (*n* = 142)	24 (51.1)	56 (58.9)	0.37
Active joint count	1 (0, 1)	1 (0, 2)	0.18
Hip arthritis (current or past) (*n* = 136)	19 (45.2)	46 (48.9)	0.69
Tarsitis (current or past) (*n* = 134)	6 (14.3)	14 (15.2)	0.89
SIJ pain with deep palpation (*n* = 127)	23 (57.5)	53 (60.9)	0.72
**Inflammatory markers (CRP or ESR, *n* = 126)**			
Abnormal	23 (18.3)	63 (50.0)	0.03
**Medication use**			
Current NSAID use	29 (61.7)	54 (56.3)	0.53
Current TNFi use	3 (6.4)	2 (2.1)	0.19
Current IL-17i use	0 0	0 0	—
Current JAKi use	0 0	0 0	—
Axial arthritis in JSpA classification score (range 0–100)	76.0 (60.0, 85.0)	79.0 (68.0, 90.0)	0.07

Sample size indicated for each variable if data incomplete.

CRP: C-reactive protein; ESR: erythrocyte sedimentation rate; IBD: inflammatory bowel disease; IL-17i: interleukin-17 inhibitor; JAKi: Janus kinase inhibitor; JIA: juvenile idiopathic arthritis; JSpA: juvenile spondyloarthritis; NSAID: nonsteroidal anti-inflammatory drug; SIJ: sacroiliac joint; SpA: spondyloarthritis; TNFi: tumour necrosis factor alpha inhibitor.

### Inflammatory MRI lesions

**Figure 2.**
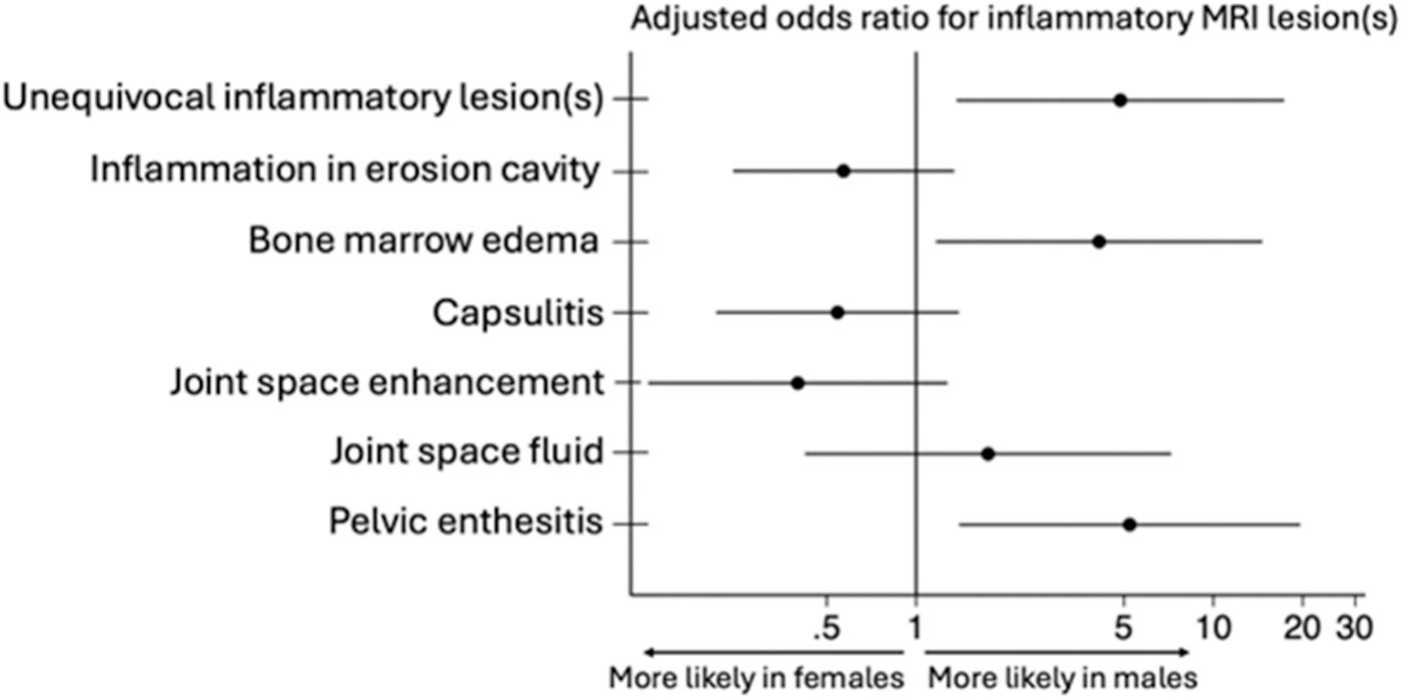
Association of unequivocal inflammatory sacroiliac joint MRI lesions typical of axial disease and sex, adjusted for age at the time axial disease was suspected

Differences in imaging characteristics between sexes are shown in Table 2. In univariate analyses, male patients with axJSpA had a significantly greater prevalence of pelvic enthesitis (*P* < 0.01), subchondral bone marrow oedema (*P* < 0.01) and unequivocal sacroiliac joint inflammation on MRI (*P* < 0.01) compared with females. After adjusting for age and inflammatory marker elevation, the odds of unequivocal inflammatory lesions typical of axial disease remained significantly higher in male patients (OR 4.86, 95% CI 1.37–17.32). In models assessing component inflammatory lesions, the adjusted odds of pelvic enthesitis outside the sacroiliac joint (OR 5.23, 95% CI 1.39–19.61) and subchondral bone marrow oedema (OR 4.13, 95% CI 1.17–14.62) remained significantly higher in males than females (Fig. 2). The results were similar when using the multiply-imputed datasets, and interaction between sex and HLA-B27 status was not significant in any of the models.

**Table 2.** Imaging features

Feature	Female (*n* = 47) N (%) or median (IQR)	Male (*n* = 96) N (%) or median (IQR)	*P*-value
Unequivocal SIJ inflammation (*n* = 141)	36 (76.6)	88 (93.6)	<0.01
Unequivocal SIJ structural lesion(s) (*n* = 140)	40 (87.0)	81 (86.2)	0.90
Subchondral bone marrow oedema	36 (76.6)	90 (93.8)	<0.01
Inflammation at site of erosion	27 (57.4)	52 (54.2)	0.71
Capsulitis	13 (27.7)	18 (18.8)	0.22
Joint space enhancement	7 (14.9)	9 (9.4)	0.33
Joint space fluid	5 (10.6)	11 (11.5)	0.88
Enthesitis outside SIJ	3 (6.4)	28 (29.2)	<0.01
Erosion	39 (83.0)	78 (81.2)	0.80
Sclerosis	18 (38.3)	48 (50.0)	0.19
Fatty lesion	6 (12.8)	12 (12.5)	0.96
Fat metaplasia in erosion cavity (backfill)	3 (6.4)	10 (10.4)	0.55
Ankylosis	0 (0.0)	4 (4.2)	0.30

Sample size indicated for each variable if data incomplete. Unequivocal SIJ inflammation defined as bone marrow oedema in ≥3 quadrants across all SIJ slices. Unequivocal SIJ structural lesion(s) defined as erosion in ≥3 quadrants or sclerosis or fat lesion in ≥2 SIJ quadrants or backfill or ankylosis in ≥2 joint halves across all SIJ slices.

JSpA: juvenile spondyloarthritis; SIJ: sacroiliac joint.

#### Structural MRI lesions

**Figure 3.**
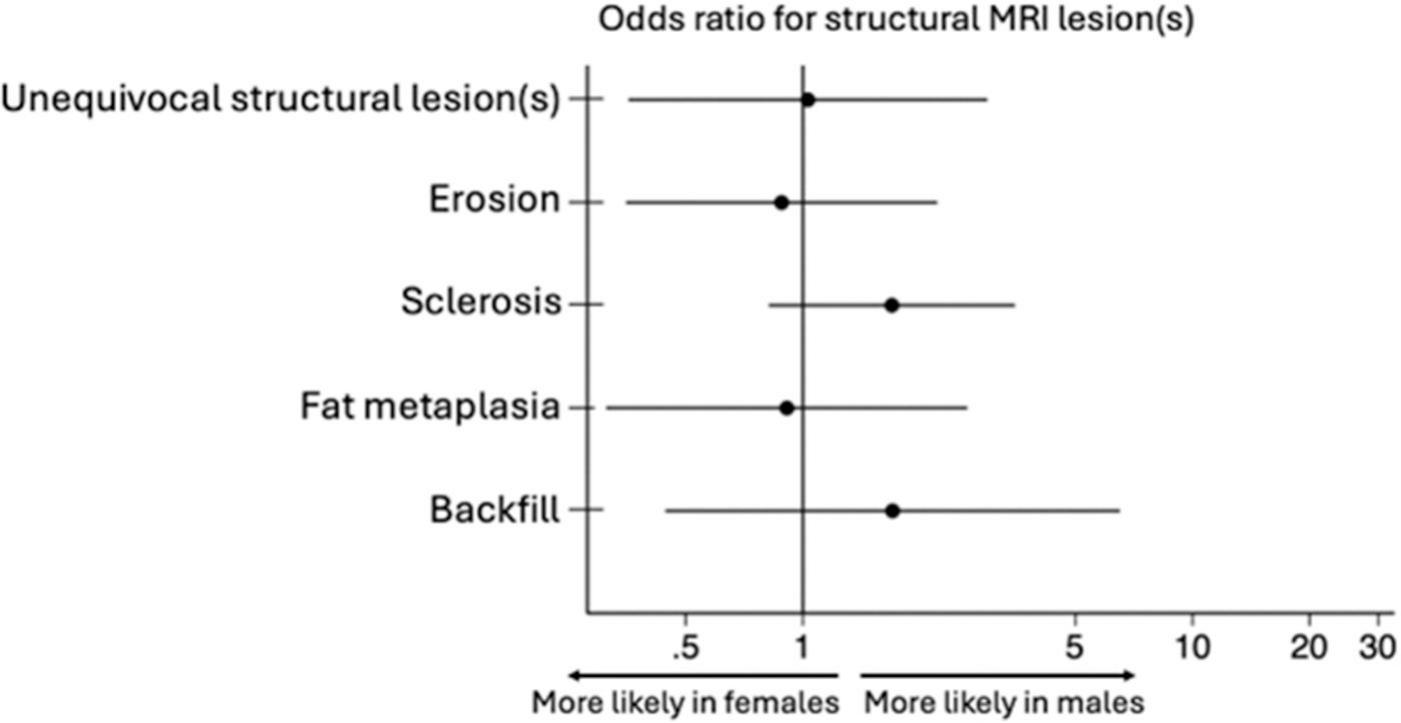
Association of unequivocal structural sacroiliac joint MRI lesions typical of axial disease and sex in univariate analyses

Univariate analyses did not reveal any significant differences between male and female patients with respect to unequivocal or component structural MRI lesions (Fig. 3). Multiple imputation did not change the results, and interaction between sex and HLA-B27 was again not significant.

## Discussion

There has been significant interest over the past decade in exploring sex differences in manifestations and outcomes of adult axSpA. This study leveraged a large international, multicentre cohort of youths with classified axJSpA to evaluate key clinical and imaging differences between male and female patients. At the time axial disease was first diagnosed, male youths with axJSpA were significantly more likely than females to have hip symptoms, HLA-B27 positive status, elevated inflammatory markers and active inflammatory sacroiliitis lesions. Patient-reported outcomes did not differ between sexes. These sex differences have important implications in the design of trials in axJSpA and offer an early window into the differences observed between male and female patients in adult axSpA cohorts.

Similar to known sex differences in adult axSpA [4, 13], male youths with axJSpA were found to have a greater burden of inflammatory sacroiliac joint lesions on MRI, CRP elevation and HLA-B27 positivity. Interestingly, despite the known association of HLA-B27 positivity with inflammatory and structural sacroiliac joint lesions in axSpA [26, 27] and radiographic progression in adult males [28, 29], HLA-B27 did not reach significance for inclusion in the inflammatory or structural sacroiliitis models or as an interaction term. Male patients with axJSpA also reported hip stiffness significantly more than their female counterparts. A Chinese retrospective cohort study of adult ankylosing spondylitis patients reported a greater degree of hip involvement at a younger age in men (median symptom onset 18 *vs* 23 years) with a similarly earlier age of total hip arthroplasty, albeit in a cohort with <10% female patients [30]. Despite the significantly higher prevalence of patient-reported hip stiffness in males in our cohort, there were no sex differences in physician-confirmed hip arthritis on exam, suggesting that the increased hip symptoms reported by male patients may be referred pain and/or stiffness from active inflammatory sacroiliitis in this group.

While several distinctions between male and female patients in this juvenile cohort are consistent with sex differences reported in the adult axSpA literature, there are also notable differences. First, despite structural sacroiliitis being more common in adult males [5, 13, 14], these differences were not observed in this study. This is possibly due to the study cohort consisting of youths with newly diagnosed axial disease and thus with less time to accrue significant structural lesions. A recent longitudinal study showed that anatomic regions of the SIJ affected by inflammatory sacroiliitis lead to local structural sacroiliitis over time [21]. Thus, it is possible that the higher degree of active inflammatory sacroiliitis in male axJSpA patients may offer an early window into the higher burden of structural sacroiliitis and radiographic progression observed in adult males [5–7, 13, 14]. Second, adult females with axSpA are additionally more likely to have worse pain scores and functional outcomes with a higher degree of tender joints and entheses [4, 7–12, 15–17, 31]. In contrast, the patient-reported pain and disease activity scores did not significantly differ between sexes in this juvenile cohort. There were also no differences in total body pain, peripheral arthritis or clinically defined enthesitis. It is possible that differences in VAS scores and joint/enthesis tenderness in adult cohorts may be biased by comorbid fibromyalgia that predominates in female patients [9, 32], particularly in cohorts of non-radiographic axSpA. Female paediatric patients more broadly with juvenile idiopathic arthritis and enthesitis-related arthritis tend to have worse pain scores [33], but this may be driven by peripheral symptoms and thus sex differences may not manifest in an axial disease cohort. Third, the prevalence of comorbid IBD and psoriasis did not differ between male and female youths despite the greater prevalence of these comorbidities in adult females with axSpA [34, 35]. This may be due to the younger median age of our cohort with less time for comorbidity development, particularly IBD which has an initial peak incidence at age 15–30 years [36, 37].

There are several strengths to this study. The study population was drawn from a large international multicentre cohort of patients who fulfilled classification criteria for axJSpA. Our findings therefore are likely generalizable to the broader axJSpA population but should not be extrapolated to patients with JSpA without axial disease. The involvement of expert radiologists in the interpretation of sacroiliac joint MRI and use of data-driven definitions of inflammatory and structural sacroiliac joint lesions enhance the validity of the outcomes explored in this study. There are also several limitations. There is a possibility of variation across centres in clinical practice around the diagnosis of JSpA and in the specific MRI protocol employed. However, these variations further enhance the generalizability of our findings to the real world where clinical practice and imaging protocols often differ between sites. This study is cross-sectional and includes patients with axJSpA at the time axial disease was initially suspected. Further study is therefore needed to determine how these sex differences may change longitudinally or in those with longer-established disease. As with any observational study there was missing data. However, this was minimal and there were no significant changes to results after multiple imputation, thus adding confidence to our findings. Active use of medications such as NSAIDs and TNFi that treat axial disease did not significantly differ between sexes and thus are unlikely to confound the observed sex differences in this cohort.

In summary, male youths with axJSpA were significantly more likely than females to have clinical and MRI features of active inflammatory sacroiliitis at the time axial disease was first diagnosed. While some differences observed between male and female youths with axJSpA mirror known differences in adult axSpA, several key distinctions from adult axSpA were found, reaffirming axJSpA as a distinct disease entity. Considering these findings, trials in axJSpA should consider stratification by sex in their study design.

## Data Availability

The data underlying this article will be shared on reasonable request to the corresponding author.
